# Dental anomalies and lesions in Eastern Atlantic harbor seals, *Phoca vitulina vitulina* (Carnivora, Phocidae), from the German North Sea

**DOI:** 10.1371/journal.pone.0204079

**Published:** 2018-10-03

**Authors:** Patricia Kahle, Catharina Ludolphy, Horst Kierdorf, Uwe Kierdorf

**Affiliations:** Department of Biology, University of Hildesheim, Hildesheim, Germany; Ecole Normale Supérieure de Lyon, FRANCE

## Abstract

Skulls of 1,901 Eastern Atlantic harbor seals (*Phoca vitulina vitulina*) were systematically studied for externally visible dental anomalies and lesions. The sample comprised 927 males and 974 female individuals, with age at death ranging between 1 week and 25 years. Most of the skulls originated from animals collected in 1988, when the population suffered from a mass mortality event caused by the phocine distemper virus (PDV). Mean age (± SD) of females (6.7 ± 6.4 years) was higher (p = 0.002) than that of males (5.9 ± 5.2 years). In 264 individuals, one or more teeth were missing either congenitally (n = 26 animals, 1.4%) or due to intravital loss (n = 238 animals, 12.5%). One male exhibited congenital absence of all teeth (anodontia). As this animal had been reported to be almost hairless, the condition was tentatively diagnosed as a case of hypohidrotic ectodermal dysplasia. Males were more frequently (p = 0.002) affected by intravital tooth loss (15.0%) than females (10.2%). Supernumerary teeth were found in 3.4% of the skulls, with females (4.7%) showing hyperodontia more frequently (p < 0.001) than males (1.9%). Fifty-nine individuals (3.1%; 28 males, 31 females, p = 0.84) exhibited abnormal tooth morphology. Tooth fractures were noted in 40 seals (2.1%), with males being more frequently affected than females (p = 0.017). Periapical lesions were diagnosed in 143 skulls, with a tendency (p = 0.05) for males (8.7%) to be more frequently affected than females (6.4%). Enamel hypoplasia was not observed in the study sample. Analyzing the occurrence of dental anomalies and lesions in wild mammals can substantially contribute to an assessment of population health and thereby broaden the basis for effective species conservation and informed management decisions.

## Introduction

Most mammals possess two successive sets of teeth (diphyodont condition), the initially formed deciduous dentition being followed by a permanent one [1–3]. Another common feature of mammalian dentitions is the functional and related morphological specialization of the teeth, a condition referred to as heterodonty [3]. Of the four different tooth types of mammals, the incisors, canines, and premolars undergo replacement, while there is only a single generation of molars [1–3]. The main functions of teeth are food acquisition and processing, but they also play a role in the social life of various mammal species [3]. Deviations from the normal number, position, morphology, or structure of teeth can negatively affect body condition, health, survival, and lifetime reproductive success of an individual [3–6].

Compared to humans and domestic animals, less is known about dental anomalies and lesions in wild mammals [7]. Several studies addressed dental variation and pathology in wild carnivorans, including different pinniped species [4–26]. Worldwide, carnivorans are threatened by human activities and their consequences, including hunting, poaching, habitat destruction, prey depletion, environmental pollution, and climate change [27, 28]. As dental anomalies or lesions may decisively affect body condition, morbidity, and mortality of wild mammals [4–7, 29], a greater knowledge of their dental pathology can considerably improve our insight into the overall health condition of populations and thereby broaden the basis for informed management decisions.

The harbor seal (*Phoca vitulina*) is the most widespread pinniped species in the northern hemisphere, with five subspecies currently recognized [30]. Of these, the Eastern Atlantic harbor seal (*P*. *v*. *vitulina*) colonizes coastal habitats and river estuaries in Western and Northern Europe [31, 32]. In 1988 and again in 2002, a phocine distemper virus (PDV)-epizootic severely reduced the population of the Eastern Atlantic harbor seal, but it quickly recovered both times [33, 34]. The total population of the Eastern Atlantic harbor seal has more recently been estimated at approximately 113,000 to 134,000 individuals [35]. Based on aerial surveys, the size of the sub-population from the Wadden Sea, to which the majority of the individuals analyzed in the present study belonged, was currently estimated at 38,100 animals [36]. In harbor seals from European coastal regions, most pups are born in late June and early July [31].

The harbor seal shows a low degree of sexual dimorphism. Adult males are slightly larger (mean body length 160 cm) and heavier (mean body mass 75 kg) than adult females (150 cm, 67 kg) [37, 38]. Males achieve sexual maturity at an age of four to five years, females at three to four years [37]. Individuals can reach an age of up to 35 years, with females, on average, living longer than males [31, 35].

The harbor seal is an opportunistic forager whose diet predominantly consists of small to medium-sized fish, cephalopods, and crustaceans. These are usually swallowed in one piece without prior chewing, and the dentition is adapted for gripping slippery prey [31, 39, 40].

**Fig 1:**
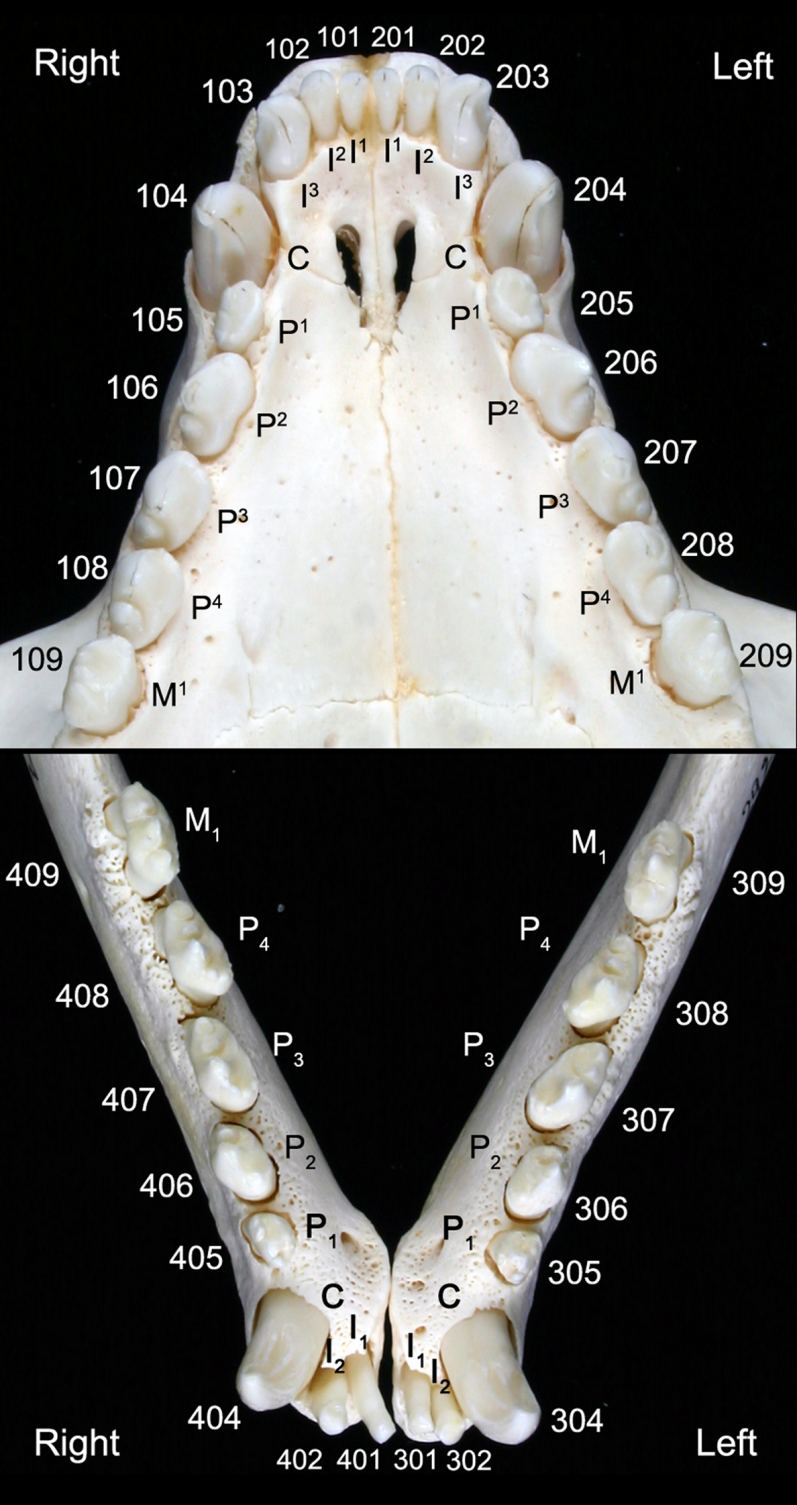
Permanent dentition of an Eastern Atlantic harbor seal (*Phoca vitulina vitulina*). The teeth in the maxillary (upper image) and mandibular (lower image) dental arcades are identified by anatomical terms and the numbering of the modified Triadan system frequently used in veterinary dentistry.

The permanent dentition of the harbor seal comprises 34 teeth (Fig 1), the dental formula being I3/2, C1/1, P4/4, M1/1 [18, 41–43]. The first and second incisors are small and peg-shaped, while the (maxillary) third incisors are larger and have a canine-like form. The well-developed canines possess large roots that extend to below the second premolar roots. The premolars and molars, often collectively referred to as postcanine teeth [43, 44], are of a relatively uniform and simple shape, with a main cusp and a varying number of smaller cusps located mesial and distal to the main cusp [43]. The first premolar is markedly smaller than the other premolars and single-rooted. Contrary to many terrestrial carnivorans, in the pinniped dentition the maxillary fourth premolar and the mandibular first molar are not differentiated into a pair of carnassials. As is typical for the Carnivora, the harbor seal has an anisognathous occlusion, the lower jaw being slightly narrower than the upper one [18].

The deciduous dentition of the harbor seal comprises 26 teeth and its dental formula is dI3/2, dC1/1, dP3/3 [45]. The number of deciduous premolars in each quadrant is three (compared to four permanent premolars), since the first premolar is present only in the permanent dentition [45]. The deciduous teeth are much smaller than their permanent successors and shed already *in utero* or shortly after birth. The permanent dentition is fully functional at the end of the short suckling period, when the young are 4 to 6 weeks old [38, 45].

Congenital deviations from the normal number, size, shape, and position of teeth mostly have genetic causes, but can also originate from a disturbance of normal development by other factors, such as disease processes, nutritional deficiencies or the impact of toxic substances [7, 46, 47]. Genetic and non-genetic developmental alterations must be distinguished from pathological changes occurring after tooth eruption. Such posteruptively acquired lesions include for instance tooth fracture, root resorption, pulpal changes, and posteruptive tooth loss [7, 46]. Finally, when studying dry skulls from scientific collections, intravital (developmental and posteruptively acquired) abnormalities and lesions must be distinguished from postmortem (artefactual) changes of the dentition occurring during preparation or handling of the skulls [18].

The aim of the present study is to provide a comprehensive overview of the spectrum and prevalence of externally visible dental anomalies and lesions in Eastern Atlantic harbor seals from the German North Sea, based on a systematic inspection of a huge number of skulls from a scientific collection.

## Materials and methods

Skulls of 1,901 Eastern Atlantic harbor seals (S1 Table), from animals collected between 1961 and 1994 at different locations along the German North Sea coast (S1 Fig), were examined. The skulls are part of the osteological collection of the Zoological Institute of the University of Kiel (ZIK), Germany. Most of the studied specimens (73.3%) were obtained in 1988 when the population was affected by a PDV-related mass mortality event, while 19.3% were collected before and 7.4% after 1988. Except for six specimens, each skull was labeled with an identification number, and all skulls had a tag attached that contained information on sex, age at death, and date and location of collection. Age-at-death determination of the seals had previously been performed based on gross-morphological criteria for individuals younger than one year and on cement-layer-analysis of canine teeth in older individuals [48]. The reconstructed period of birth of the seals (based on the age-at-death data) ranged between 1951 and 1994. The cause of death of the animals is mostly not recorded on the tags. The majority of animals collected in 1988 were, however, most likely victims of the PDV-epizootic. Fifteen of the individuals from 1988 were young-of-the-year, i.e., they were born in that year. Of the animals collected between 1989 and 1994, six had been born in 1988. This means that 21 harbor seals had grown their teeth in the year of the PDV-outbreak.

All skulls were inspected for congenital and posteruptively acquired dental anomalies and lesions, including signs of periapical processes. Some specimens were viewed under a digital reflected-light microscope (Keyence VHX-500F, Keyence Corp., Osaka, Japan) equipped with a high performance zoom lens (Keyence VH-Z20R; magnification range 20-fold to 200-fold). In a few cases, radiographs were taken in order to improve the diagnosis.

The dentition and the tooth-bearing bones were examined systematically based on established criteria [13, 18]. Initially, the dentition was assessed for completeness, and deviations from the regular number of teeth (due to supernumerary or missing teeth) were determined. In the case of supernumerary teeth, their shape and the position in the tooth row were recorded. Postmortem tooth loss (during preparation or handling of the skulls) was diagnosed when an alveolus was open (no obliteration) with well-defined and sharp alveolar margins. Partial or complete filling of an (often widened) alveolus with spongious bone (re-ossification) was regarded as evidence for intravital tooth loss. In these cases, the alveolar bone was frequently reduced in height and the alveolar margins were rounded, indicating tooth loss due to advanced periodontitis [7, 13].

We further recorded abnormal tooth positions, abnormal shapes of crown and/or root, and deviations from the normal number of roots. Tooth fusion was distinguished from gemination (incomplete division of a tooth germ) based on the number of teeth in the dentition [47]. In the case of fusion, this number is decreased by one, as there is a single, abnormally formed fusion product instead of two separate teeth. In contrast, in the case of gemination the number of teeth is normal but one of them exhibits an abnormal shape. When teeth had been glued into their alveoli or could not be removed due to snug fit, the number of roots was determined by inspecting the visible coronal root portion. All skulls were inspected for the presence of persistent deciduous teeth that due to their smaller size can be easily distinguished from permanent teeth.

Tooth fractures were classified according to the American Veterinary Dental College classification system [49]. We distinguished between enamel fracture (loss of enamel only), uncomplicated crown and crown-root fracture (no opening of the pulp cavity), complicated crown and crown-root fracture (with opening of the pulp cavity) and root fracture. In crown fractures, dentin and enamel were affected, in crown-root fractures in addition also the cementum. We attempted to clearly differentiate between intravital fractures/loss of tooth substance (the edges of the defects were rounded to different extent) and postmortem breakage and chipping (sharp-edged defects).

The skulls were also examined for signs of periapical lesions. On external inspection, early stages of such lesions (smaller lytic cavities around the root tip) could not be identified. The diagnosis of periapical lesions was thus limited to more advanced cases, in which openings of draining tracts were present in the jaw bones or where these bones exhibited other indications of an underlying inflammatory process, e.g. high number of vascular foramina or focal apposition of periosteal new bone. Care was taken to distinguish fenestrations (openings of draining tracts) in the jaw bones from larger regular foramina that can vary individually in number, size and position [50].

Furthermore, all teeth were macroscopically inspected for the occurrence of enamel hypoplasia, i.e., for developmental defects (grooves, pits, or plane-type defects) in the enamel surface resulting from a disturbance of enamel matrix secretion [51–53].

For analysis, the individuals of the study sample were grouped into the following three age classes: (a) “neonatal/early postnatal” (0 to 4 weeks of postnatal age), (b) “juvenile/subadult” (>4 weeks to 5 years), and (c) “adult” (>5 years). This classification is based on life history events of the study species, *viz*., the age at weaning (4 to 6 weeks) [38] and the age at attaining physical and sexual maturity (about 5 years) [31].

Given the number of inspected skulls and assuming a normal number of 34 teeth for all studied individuals, the potential total maximum (PTM) number of teeth available for analysis was 64,634. A full complement of teeth in all individuals was assumed when calculating the frequency of numerical dental anomalies.

Differences in the occurrence of dental anomalies and lesions between sexes within age classes and for the total study sample were tested using Pearson’s Chi-squared test. The same test was also used to analyze differences in the frequency of dental anomalies and lesions between age classes and anatomical locations. The t-test for independent samples was used to test for age-at-death differences between males and females. In all tests, p-values < 0.05 were considered to indicate statistical significance.

No specific permits were required for the present study, which was performed on archived specimens from a scientific collection that had not been specifically obtained for the purpose of this investigation. Specimen collection and curation were conducted in accordance with all relevant institutional, national and international guidelines.

## Results

### Sex and age distribution

**Fig 2:**
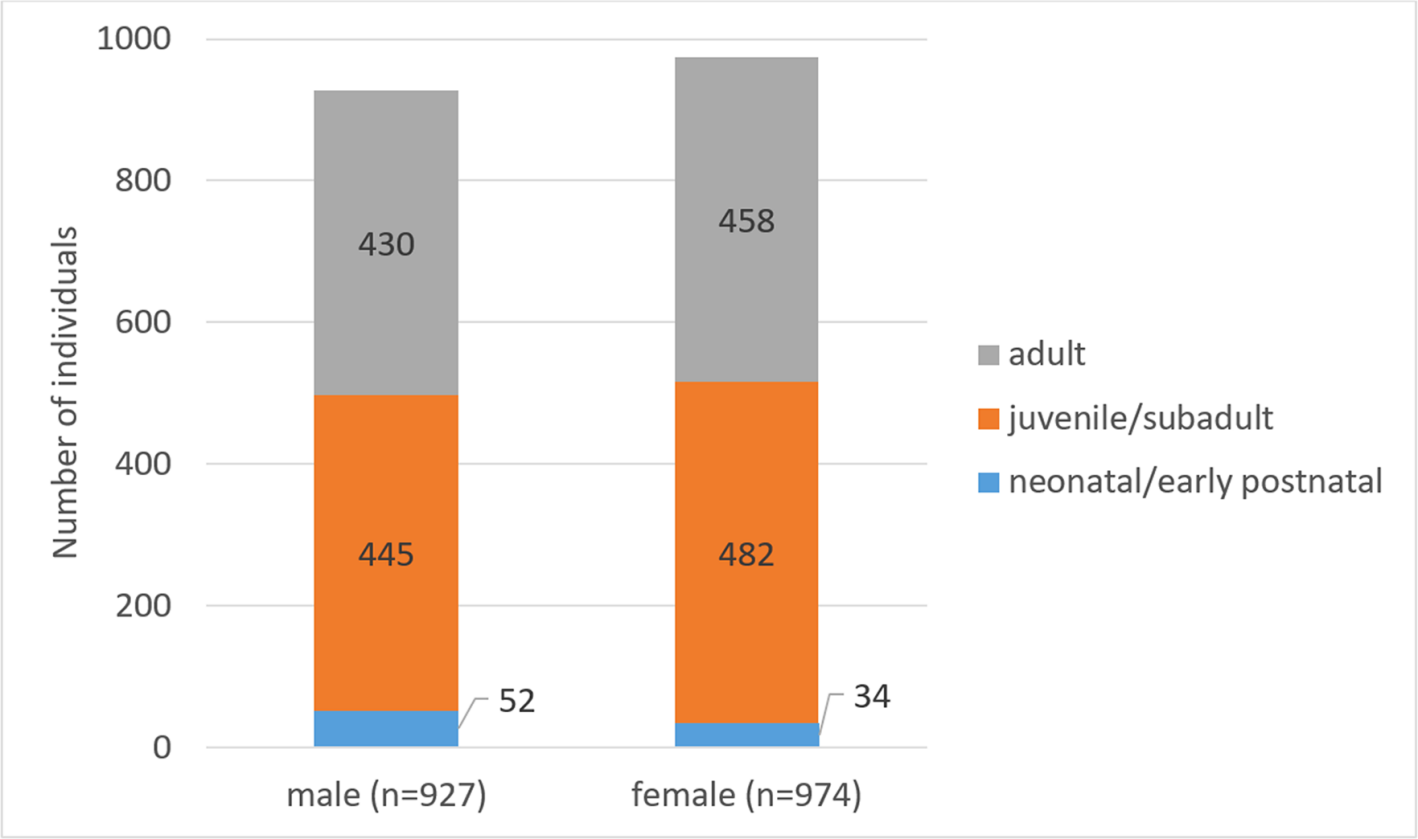
Overview of examined Eastern Atlantic harbor seals (*Phoca vitulina vitulina*). Numbers of males and females in three age classes are given.

Of the 1,901 examined skulls, 927 (48.8%) belonged to male and 974 (51.2%) to female harbor seals (Fig 2). Neonatal/early postnatal, juvenile/subadult, and adult specimens accounted for 4.5%, 48.8% and 46.7%, respectively, of the total number of individuals (for absolute numbers see Fig 1). Mean age (± SD) of females (6.7 ± 6.4 years) was significantly (p = 0.002) higher than that of males (5.9 ± 5.2 years). Maximum age was 25 years for both sexes.

### Missing teeth

**Fig 3:**
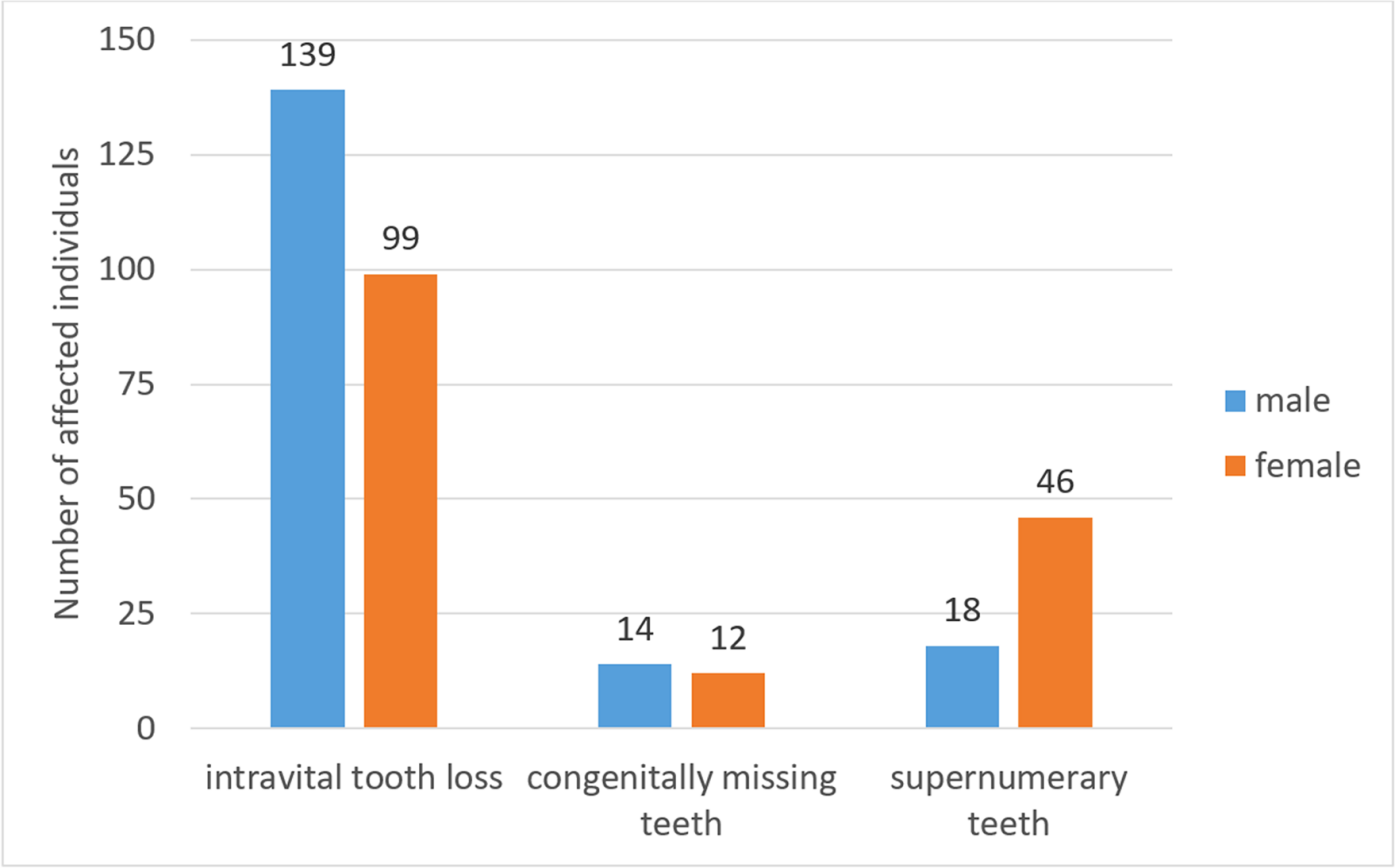
Numbers of Eastern Atlantic harbor seals (*Phoca vitulina vitulina*) in the study sample exhibiting numerical dental anomalies. The criteria for classifying cases of missing teeth are given in the text.

In 264 harbor seals, one or more teeth were missing in the dentition either congenitally (tooth agenesis; 26 individuals, 1.4% of examined seals) or due to intravital loss (238 individuals, 12.5% of examined seals) (Fig 3). The number of teeth (supernumeraries excluded) present for evaluation in the skulls was 60,775 (94% of PTM). Postmortem loss (artefactual absence) was diagnosed for 3,085 teeth (4.8% of PTM), intravital loss for 708 teeth (1.1% of PTM), and 66 teeth (0.1% of PTM) were missing congenitally.

**Fig 4:**
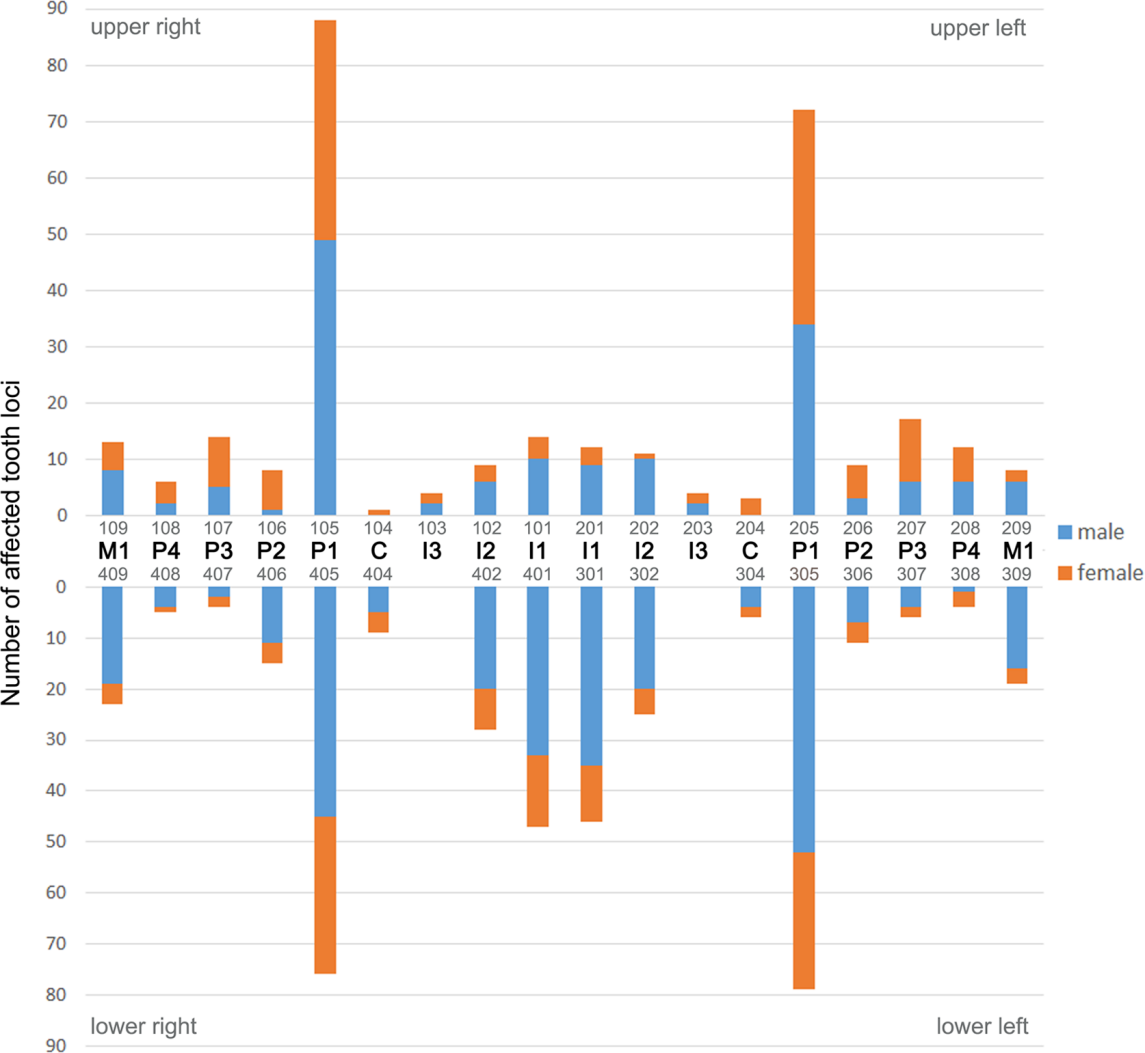
Distribution of intravital tooth loss in the study sample of Eastern Atlantic harbor seals (*Phoca vitulina vitulina*). Absolute numbers for the different tooth loci are given. The teeth are identified both by anatomical terms and the numbering of the modified Triadan system (see Fig 1).

Except for three cases in animals from the age class juvenile/subadult, all intravital tooth losses were recorded in adult individuals. Males (139/927, 15.0%) were significantly more frequently (p = 0.002) affected by intravital tooth loss than females (99/974, 10.2%). Of the 708 intravitally lost teeth, 403 (56.9%) were missing in the mandibular and 305 (43.1%) in the maxillary dentition (p < 0.001). There was no significant difference between sides for intravital tooth loss, 364 cases (51.4%) occurring in the right half and 344 cases (48.6%) in the left half of the dentition (p = 0.45). The first premolars were the teeth most often lost intravitally (315 of 708 cases, 44.5%). The distribution of intravital tooth loss in the dentition is illustrated in Fig 4.

**Fig 5:**
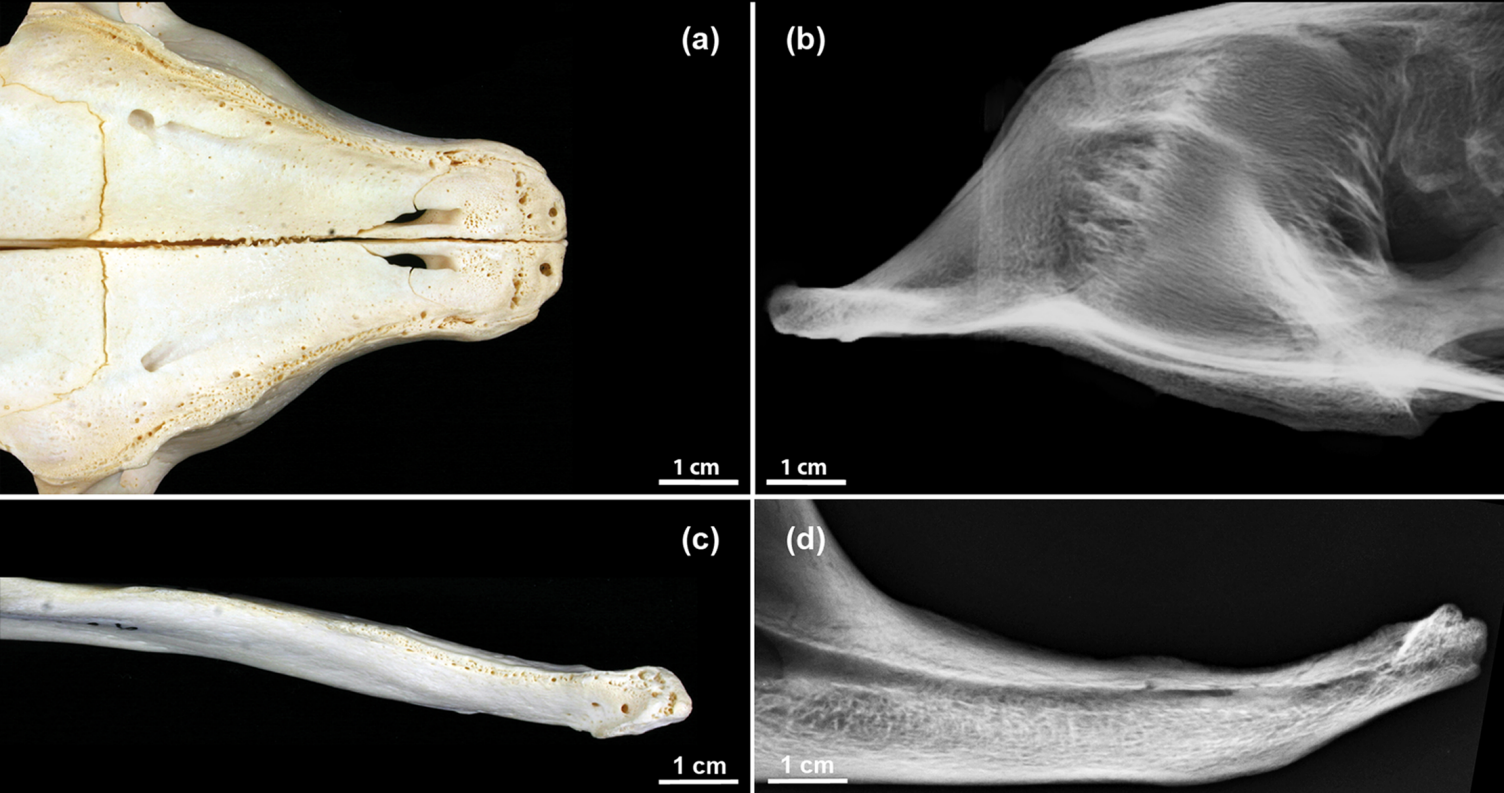
Skull of an anodont 5-year-old male Eastern Atlantic harbor seal (*Phoca vitulina vitulina*, specimen ZIK 4626). **(a)** Ventral view of incisive and maxillary bones. **(b)** Radiograph of anterior skull region (laterolateral projection). **(c)** Dorsal view of left mandible. **(d)** Radiograph of left mandible (mediolateral projection). Note that the (tooth-dependent) alveolar structures are also missing.

Of the 26 seals exhibiting congenital absence (agenesis) of teeth, one male individual (specimen ZIK 4626) was completely toothless (anodont condition) (Fig 5). According to a short note published on the animal while it was still alive [54], it had been found in the wild in July 1958 at an age of about two weeks. The individual was transferred to the zoo in Bremerhaven, where it lived until its death at an age of five years. The skeleton was added to the collection of the Zoological Institute of the University of Kiel. The short note also mentions that, except for a few patches of hair in the head, neck, and shoulder regions, the animal had been hairless. A diagnosis of the condition was, however, not put forward. The right mandible, which according to a note on the tag had been fixed in formalin, could not be located by us. The complete lack of teeth in the individual had, however, previously been confirmed radiographically in the living animal [54]. Our macroscopic and radiographic inspection showed that alveolar structures were also completely missing in the individual (Fig 5).

**Fig 6:**
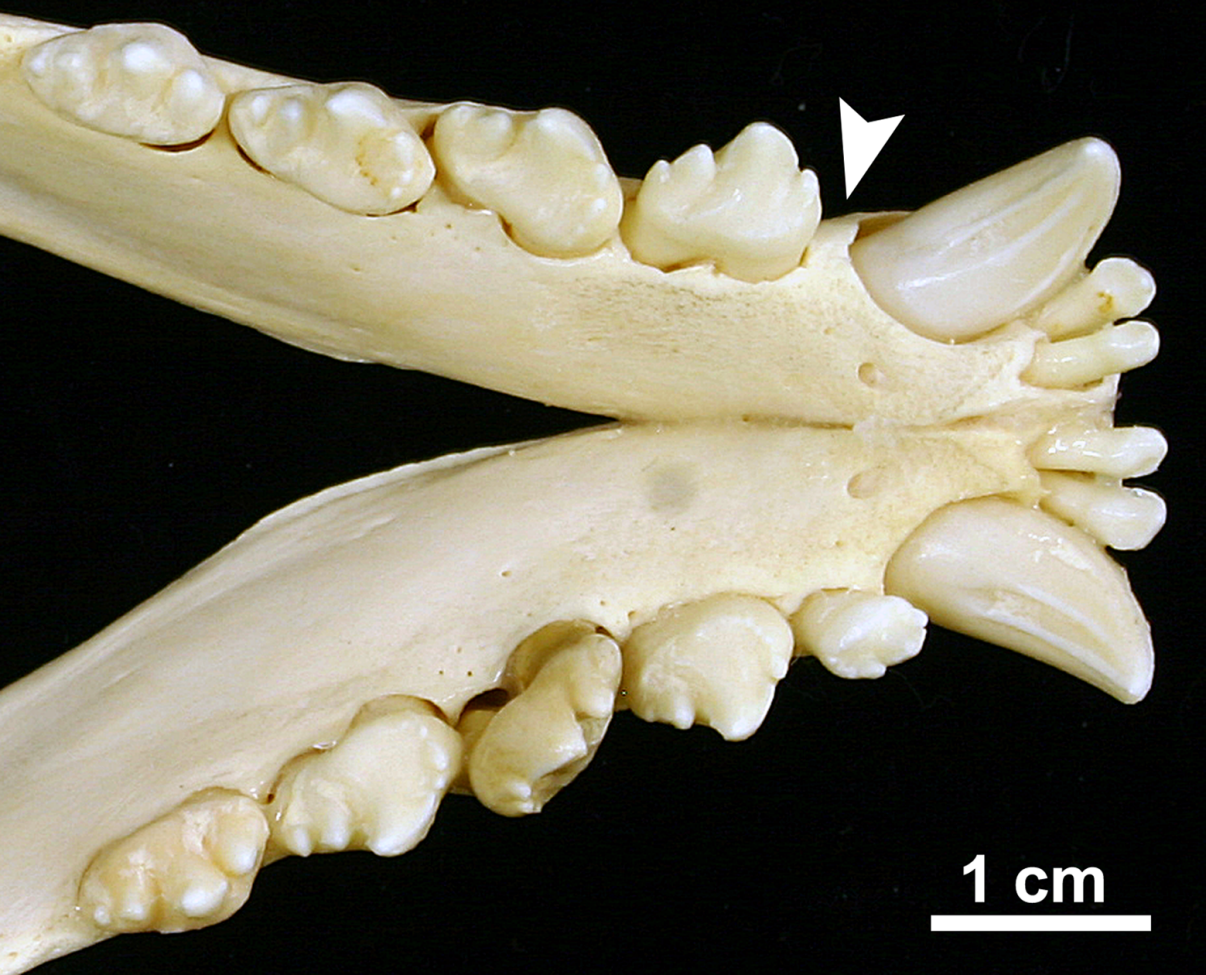
Example of congenital tooth absence in an Eastern Atlantic harbor seal (*Phoca vitulina vitulina*). Dorsal view of the mandibles of a 1-year-old male (specimen ZIK 24885). The left P_1_ is missing, there is no indication of an alveolus (arrowhead), and the distance between canine and P_2_ is shorter on the left than on the right side.

The remaining 25 seals (13 males, 12 females) exhibited 32 cases of congenital tooth absence, which can all be classified as examples of hypodontia, i.e., agenesis of only a few teeth. One individual lacked 5 teeth (all four first premolars and the right M^1^), three individuals each lacked two teeth in a bilaterally symmetrical fashion (1 × both I^1^, 2 × both P_1_) and in each of the remaining 21 seals, a single tooth was missing. Six of the 32 cases (18.8%) occurred in the maxillary and 26 (81.3%) in the mandibular dentition. Right side (15 cases, 46.9%) and left side (17 cases, 53.1%) were almost equally affected by congenital absence of teeth. The tooth position most frequently affected by agenesis (anodont specimen excluded) was the mandibular first premolar (13 of 32 cases, 40.6%) (Fig 6), followed by the mandibular first molar (6 cases, 18.8%) and the mandibular first incisor (4 cases, 12.5%). The other tooth positions for which agenesis was recorded were I^1^ (3 cases), I_2_ (3 cases), P^1^ (2 cases) and M^1^ (one case). The difference between the sexes in the occurrence of hypodontia/anodontia was not significant (p = 0.60).

### Supernumerary teeth

**Fig 7:**
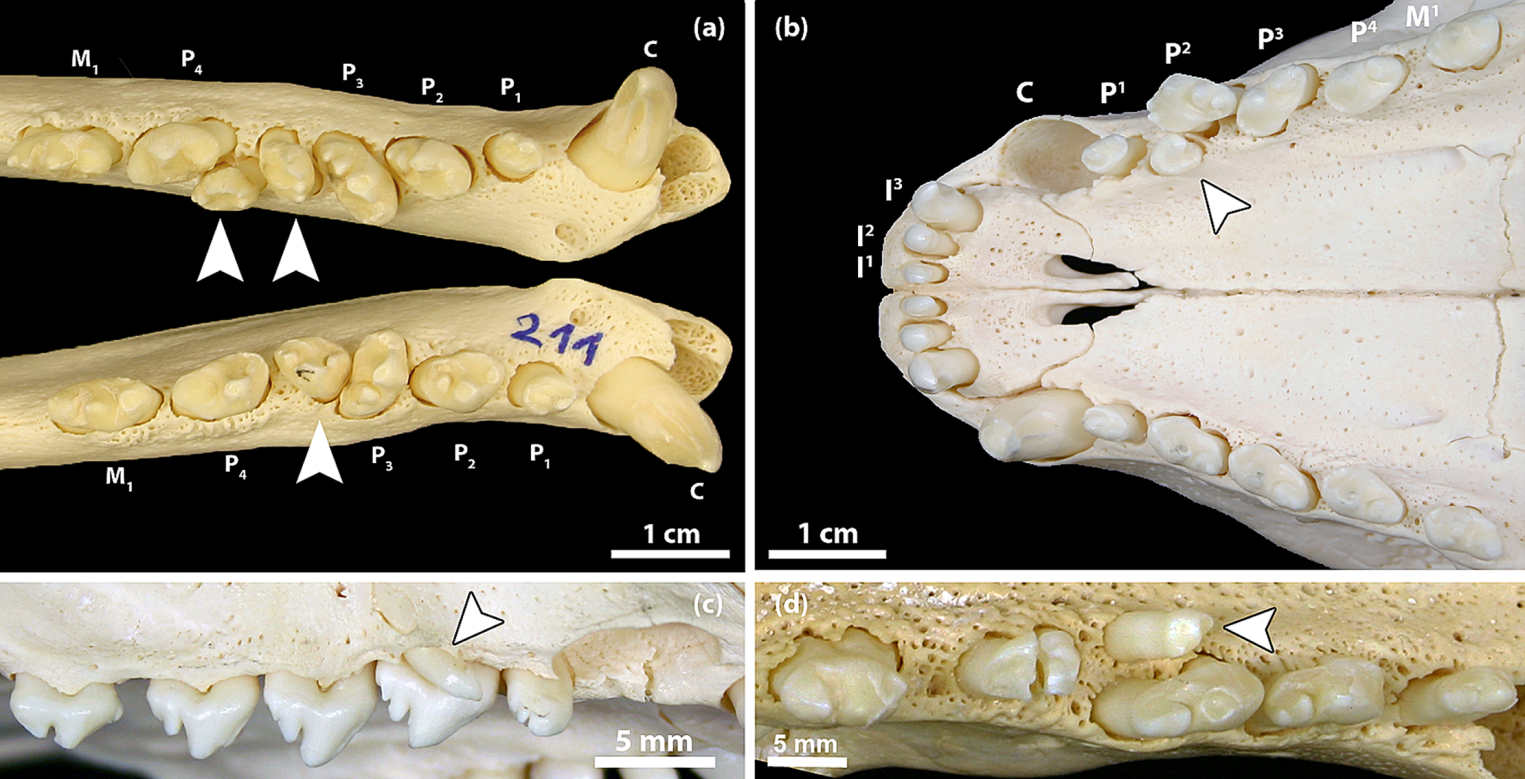
Examples of supernumerary teeth in the permanent dentition of Eastern Atlantic harbor seals (*Phoca vitulina vitulina*). **(a)** Dorsal view of the mandibular tooth rows of a 2-year-old female (specimen ZIK 24806). There are two supernumerary teeth (located between P_3_ and P_4_) in the left and one supernumerary tooth in a corresponding position of the right mandible (arrowheads). Due to the formation of the supernumeraries, both teeth at the normal P_3_ position are rotated approximately 45 degrees. The condition is interpreted as the result of a splitting of both the P_3_ and the P_4_ tooth germs on the left side and of the P_3_ tooth germ on the right side. **(b)** Ventral view of maxillary and incisive bones of a 3-year-old female (specimen ZIK 28942) showing a (two-rooted) supernumerary tooth (arrowhead) situated palatal to the left P^2^. The cause of the hyperodontia is most probably a splitting of the P^2^ tooth germ. **(c)** Lateral view of the right maxilla of a 1-year-old male (specimen ZIK 28170), showing a peg-shaped and mesially inclined supernumerary tooth (arrowhead) located buccal to the P^2^ (mesial to the right). **(d)** Ventral view of left maxilla of a 10-year-old female (specimen ZIK 29485), showing a peg-shaped, mesially inclined supernumerary tooth (arrowhead) located palatal to the P^3^ (mesial to the right).

Supernumerary teeth were recorded in 64 individuals (3.4% of examined seals) (Fig 3). Females (46/974, 4.7%) were significantly more frequently (p < 0.001) affected by hyperodontia than males (18/927, 1.9%). The total number of supernumerary teeth was 81. Of these, 74 (91.4%) were present in the mandibular and 7 (8.6%) in the maxillary dentition. Forty-nine animals exhibited only a single supernumerary tooth, thirteen animals had two bilaterally symmetrical supernumerary teeth, and two individuals in addition possessed an additional (third) supernumerary tooth (Fig 7A–7D).

Most of the supernumerary teeth (96.3% of all cases) were associated with the third and fourth premolars (Fig 7A). The additional tooth was often located between the third and the fourth premolar, and it could sometimes not be decided which of the two teeth had been duplicated. The supernumerary tooth was frequently located buccal or lingual (palatal) to the tooth row, and the neighboring teeth were often rotated or slightly displaced (Fig 7A). The supernumerary teeth were relatively similar in shape but mostly smaller than the adjacent teeth (Fig 7A and 7B). However, two peg-shaped supernumerary teeth were found in the maxillary premolar region of two individuals (Fig 7C and 7D). Occurrence of molars located distal to the first molars was not observed in the study sample.

### Persistent deciduous teeth

None of the 1,901 examined skulls exhibited persistent deciduous teeth. In one individual (specimen ZIK 26287, two-week-old female), some shed deciduous teeth were found that still adhered to remnants of dried soft tissue covering the jaws. The roots of the deciduous teeth showed signs of resorption. The individual had a complete set of permanent teeth, root formation of which was either already well advanced (incisors and canines) or had just begun (premolars and molars).

### Abnormal tooth morphology

**Fig 8:**
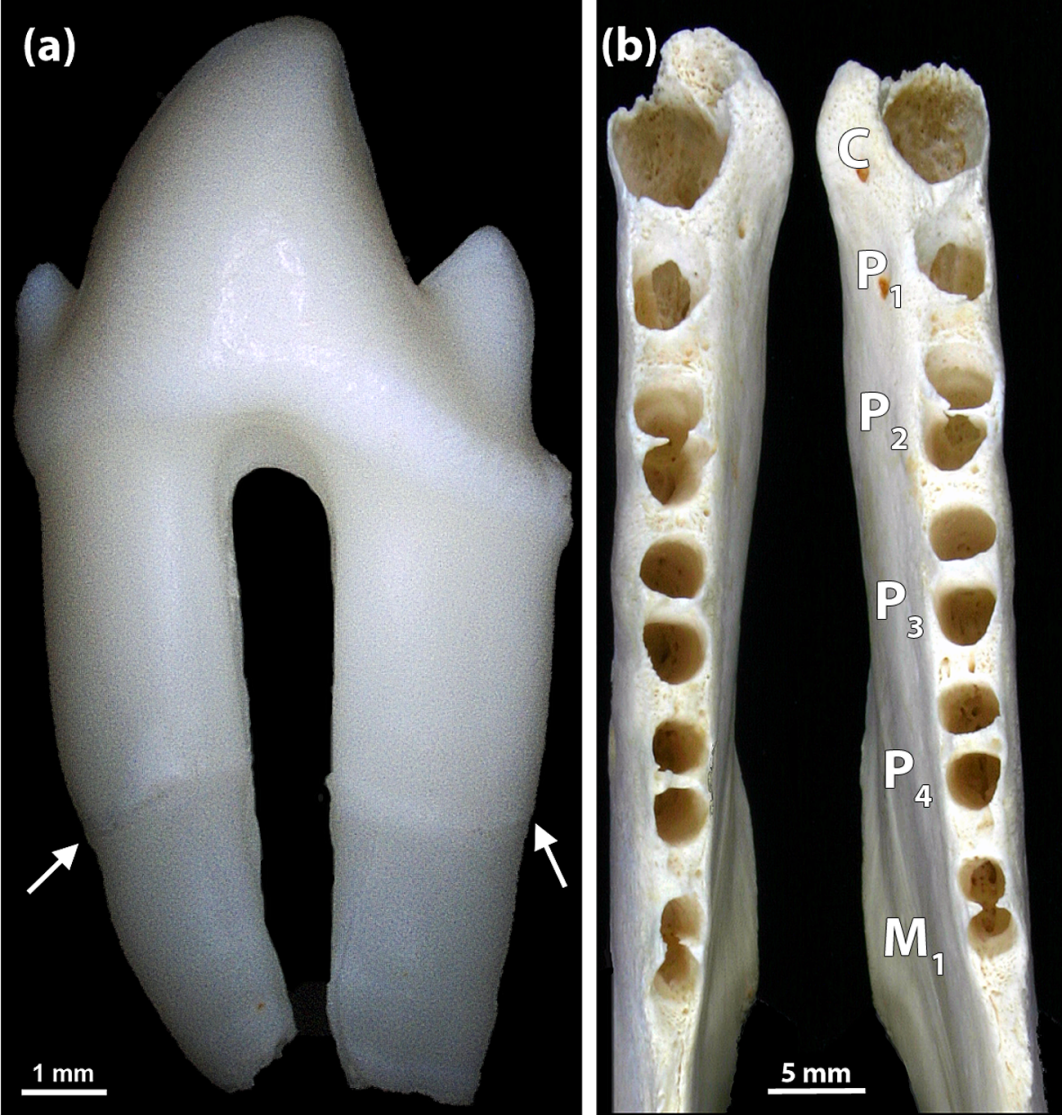
Simplified crown shape and elongated tooth roots in a 2-week-old male Eastern Atlantic harbor seal (*Phoca vitulina vitulina*, specimen ZIK 27251). **(a)** Premolar with simplified (tricuspid) crown and elongated roots. Arrows: periradicular bands. **(b)** Dorsal view of the mandibles, with identification of the alveoli for the canine, the premolars and the M_1_. Note abnormally wide interdental septa.

Fifty-nine skulls (3.1% of the study sample; 28/927 males, 3.0%; 31/974 females, 3.2%; p = 0.84) exhibited abnormalities in tooth morphology. One 2-week-old male (specimen ZIK 27251) showed abnormally elongated roots on all premolars and molars present and occurrence of (multiple) periradicular bands on all teeth (n = 23) available for inspection (Fig 8), the latter feature being indicative of a growth disturbance from a systemic cause. Furthermore, the premolars and molars were smaller than normal (except for root length) and exhibited a simplified, tricuspid crown morphology with two small cusps located, respectively, mesial and distal to a large central one. Root formation in the teeth of the individual was abnormally advanced for its young age. It could be speculated that reduced size and advanced root formation are developmentally related phenomena. Due to the small tooth size, the interdental septa between the alveoli of the premolars and molars were wider than normal. Incisors were not available for study, as the incisive bones were missing (lost during preparation of the skull) and the mandibular incisors had also been lost postmortem.

**Fig 9:**
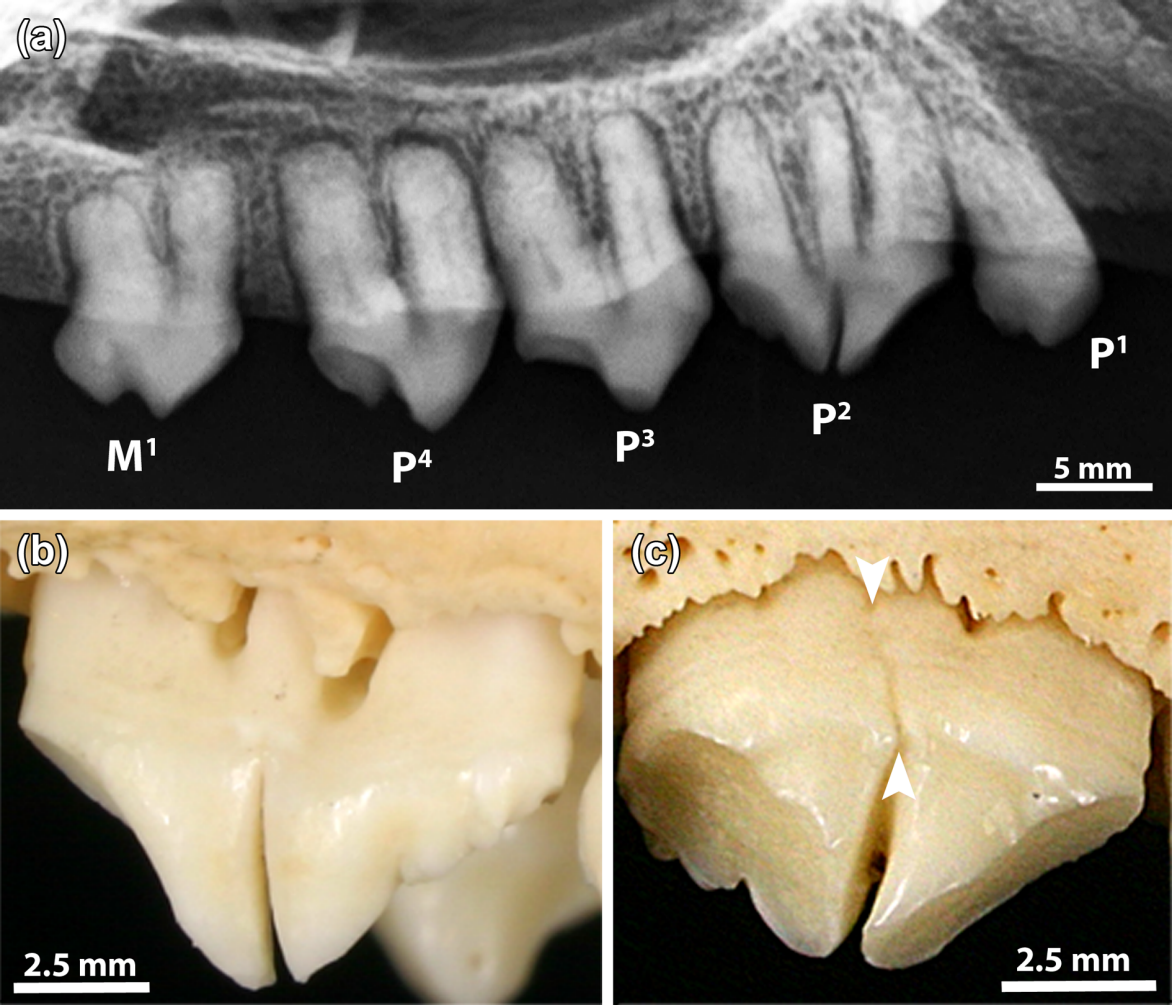
Abnormal crown and root morphology in an 11-year-old female Eastern Atlantic harbor seal (*Phoca vitulina vitulina*, specimen ZIK 29115), diagnosed as a case of gemination. **(a)** Radiograph (mediolateral projection) of left maxillary postcanine tooth row. Note bifid crown and occurrence of three roots in the P^2^, and presence of a small supernumerary root arising from the bifurcation area in the P^3^. **(b)** Buccal view of left P^2^. Buccally, the notch in the tooth crown does not extend onto the central root. **(c)** Palatal view of left P^2^. Palatally, the notch extends onto the central root. Arrowheads: vertical furrow on the central root.

**Fig 10:**
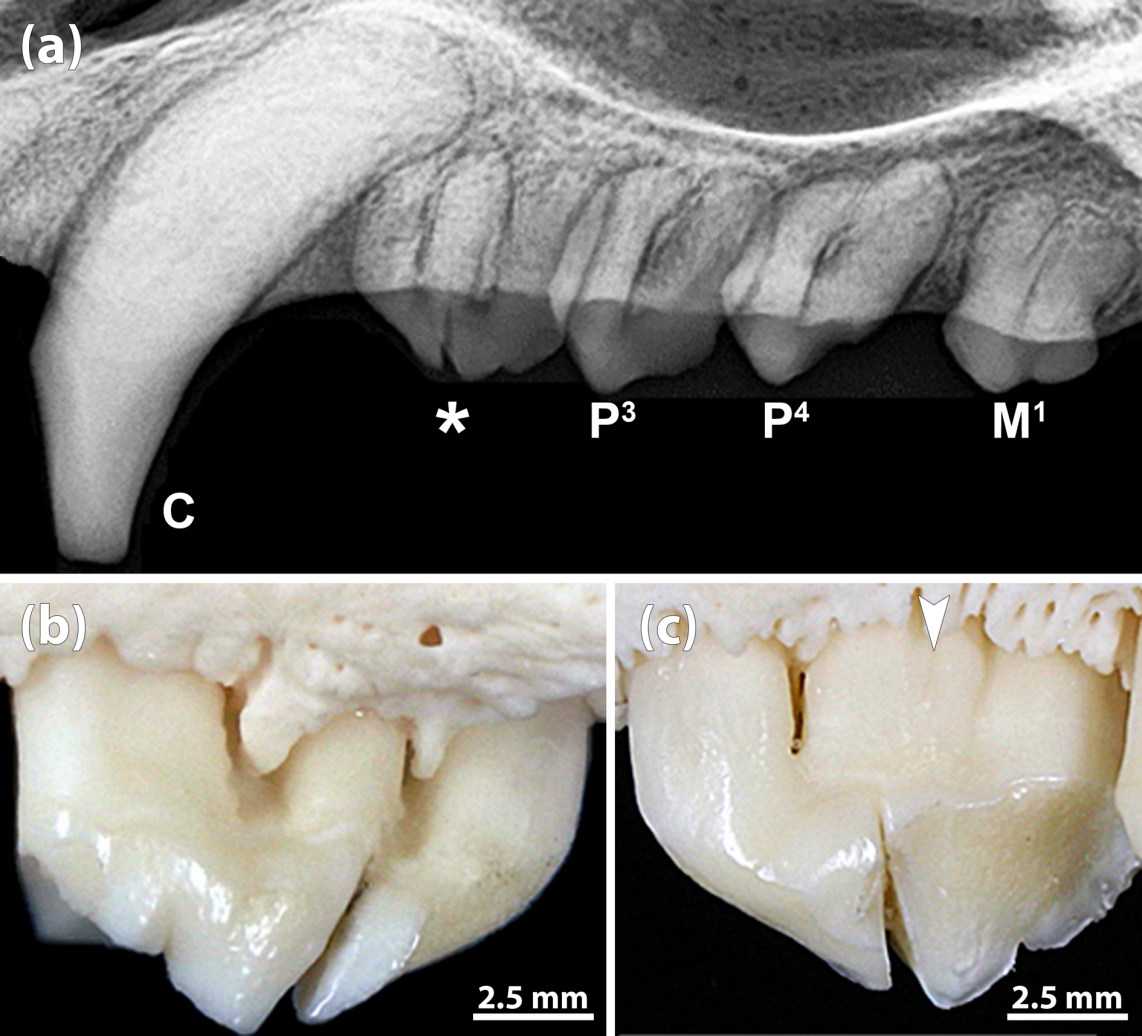
Abnormal crown and root morphology in an 18-year-old male Eastern Atlantic harbor seal (*Phoca vitulina vitulina*, specimen ZIK 29091), diagnosed as a case of incomplete fusion of P^1^ and P^2^. **(a)** Radiograph (mediolateral projection) of maxillary right canine and postcanine teeth. Asterisk: incompletely fused right P^1^ and P^2^. Note hypercementosis on the roots of the postcanine teeth. **(b)** Buccal view (mesial to the right) of incompletely fused right P^1^ and P^2^ (crowns separate). **(c)** Palatal view (mesial to the left) of incompletely fused right P^1^ and P^2^. Arrowhead: shallow furrow on the central root.

**Fig 11:**
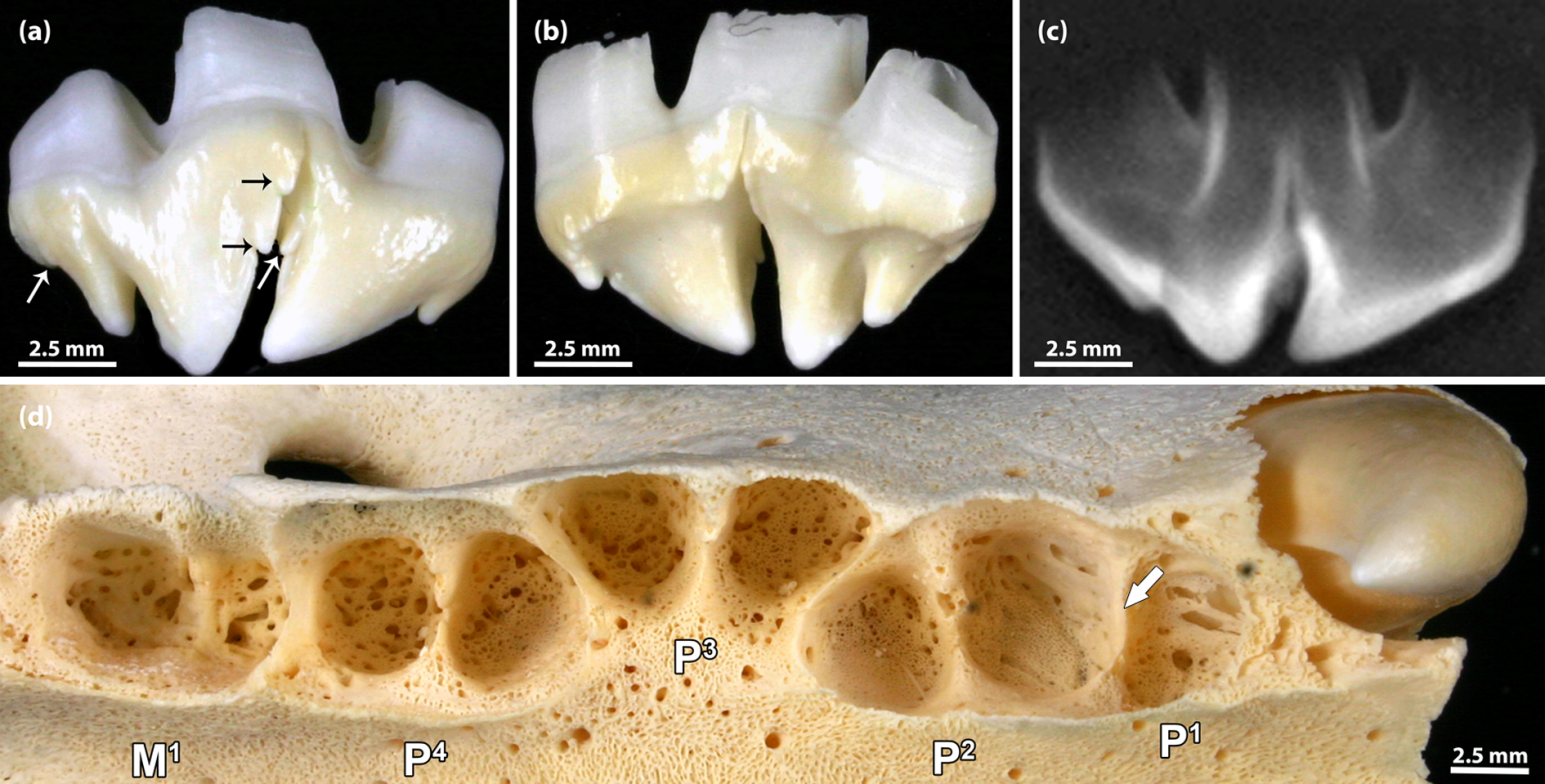
Incompletely fused right P^1^ and P^2^ in a 1-month-old male Eastern Atlantic harbor seal (*Phoca vitulina vitulina*, specimen ZIK 27291). **(a)** Buccal view (mesial to the right) of the incompletely fused teeth (crowns separate, only roots fused). Note small cusps (some marked by arrows) at the mesial and distal crown edges of both teeth. **(b)** Palatal view of the incompletely fused teeth (mesial to the left). **(c)** Radiograph of the specimen, showing that the crowns of the two teeth are separate and only the roots are partly fused. **(d)** Alveolar pattern of right maxillary postcanines. The number of alveoli is normal, as the fused root is situated in the anterior (mesial) socket of the P^2^. The septum marked by the arrow must be classified as an interradicular septum rather than an interdental septum normally present in this location.

Three seals exhibited a three-rooted (instead of the normal two-rooted) maxillary premolar with an aberrant crown shape at the P^2^ position or the P^1^ and P^2^ positions, either on the left side (adult female, specimen ZIK 29115) or the right side (adult male, specimen ZIK 29091; and one-month-old male, specimen ZIK 27291). In all three cases, the tooth crown was abnormally long and bifid (Figs 9–11).

In specimen ZIK 29115, the crown halves were of nearly equal size and shape (Fig 9A–9C). The supernumerary (third) root was located underneath a vertical notch in the tooth crown. Palatally, this notch extended as a furrow onto the root (Fig 9C). As the number of teeth in the left maxillary tooth row was normal, the abnormality was diagnosed as representing an example of gemination, i.e., to be the result of an incomplete splitting of the P^2^ tooth bud.

In specimen ZIK 29091, the premolar tooth crown also exhibited a notch that did, however, not extend onto the root (Fig 10A–10C). As the number of teeth in the right maxillary tooth row was reduced by one, the abnormality was diagnosed as a case of incomplete fusion of P^1^ and P^2^. The radiograph showed hypercementosis on the roots of all four postcanine teeth of the right maxillary tooth row (Fig 10A).

In specimen ZIK 27291, the number of teeth in the right maxillary tooth row was also reduced by one, and the macroscopic and radiographic findings (Fig 11A–11C) demonstrated another case of incomplete fusion (roots only) of P^1^ and P^2^. The two (unfused) crown portions showed the typical small cusps at their mesial and distal edges (Fig 11A and 11B). As is evidenced by the radiograph (Fig 11C), the central root of the tooth specimen was composed of two fused components, formed, respectively, by the first and the second premolar. While the maxillary second premolar is typically two-rooted, the first premolar is single-rooted. The number of alveoli for the right maxillary postcanine teeth was normal (Fig 11D), as the fused root was located in the alveolus normally holding the mesial root of the P^2^.

In seven skulls, crown and root fusion of the two mandibular incisors (I_1_ and I_2_) was noted. In two of these skulls, the anomaly occurred bilaterally, while in the remaining five it occurred unilaterally (4 cases in the left and one case in the right mandible). In all cases, the incisal crown portions of the fused incisors had remained separate. Two other individuals exhibited bilateral connection of only the roots of the mandibular incisors. As we did not obtain radiographs of the specimens, it cannot be decided whether these were cases of fusion (involving confluence of dentin) or concrescence (connection by cementum only). Four seals showed connection of the two roots of a premolar by a sheet of cementum (n = 5 teeth).

In addition to the individual with elongated roots combined with small-sized tooth crowns (specimen ZIK 27251) described above, two other cases of abnormal root shape were observed. In one case, the root of the right maxillary first premolar was bent distally at an angle of about 45 degrees. The other case was a supernumerary right mandibular premolar (situated between P_3_ and P_4_), whose distal root was much smaller than the mesial one.

Supernumerary roots were observed in 47 teeth from 40 individuals (16 males, 24 females). There was no significant difference between the sexes in the occurrence of the condition (p = 0.26). The maxillary first molar was the tooth most frequently showing a supernumerary root (83.0% of all affected teeth). The remaining cases were observed in maxillary and mandibular premolars. In six seals, both M^1^s had a supernumerary root, two individuals possessed an M^1^ with two supernumerary roots, and one individual showed a supernumerary root on both P^2^. Twenty-three supernumerary roots were situated buccally, 23 lingually, and three centrally between the two main roots. Typically, the supernumerary roots were smaller than the regular ones.

### Tooth fractures

**Fig 12:**
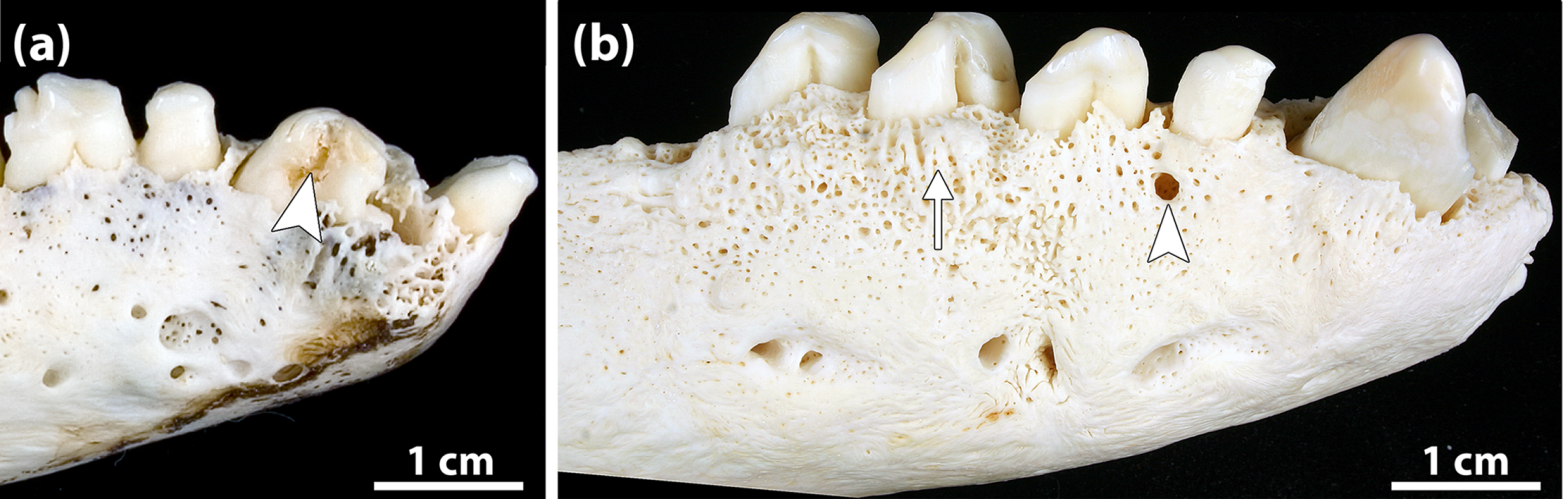
Examples of tooth fracture and sign of periapical lesion in Eastern Atlantic harbor seals (*Phoca vitulina vitulina*). **(a)** Fractured right mandibular canine of a 22-year-old female (specimen ZIK 28104). This is a case of complicated crown fracture as the pulp cavity has been opened (arrowhead). The right I_2_ had been lost intravitally. Also note signs of advanced periodontitis (porosity of alveolar bone due to numerous vascular foramina, and horizontal and vertical loss of alveolar bone) and pronounced signs of wear on the teeth. **(b)** Right mandible of a 23-year-old male (specimen ZIK 28745). The alveolar bone shows a focal osteolytic area, diagnosed as the opening of a draining tract for pus discharge. This is considered indicative of a periapical lesion associated with the P_1_. The alveolar bone shows signs of advanced periodontitis in the form of porosity of the alveolar bone (denoting increased vascularity), alveolar exostosis (arrow), and loss of alveolar bone. Note also pronounced wear signs on the teeth.

Tooth fractures were noted in 40 seals (2.1% of the study sample), with males (27/927, 2.9%) being significantly (p = 0.017) more often affected than females (13/974, 1.3%). In total, 55 teeth (0.1% of the teeth available for study) were fractured, with 52.7% of the fractures affecting maxillary and 47.3% mandibular teeth. A significantly (p = 0.007) higher proportion of adults (28/888, 3.2%) than juveniles/subadults (12/927, 1.3%) exhibited at least one fractured tooth. No dental fractures were found in neonatal/early postnatal seals. The canine was the most frequently affected tooth type (43.6% of all fracture cases) (Fig 12A). All types of tooth fractures were observed, the most frequent one being complicated crown fractures (40.0% of recorded cases), followed by uncomplicated crown fractures (36.4%) and complicated crown-root fractures (12.7%).

### Periapical lesions

In 143 of the 1901 examined skulls (7.5%) at least one periapical lesion was diagnosed (Fig 12B). There was a tendency for males (81/927, 8.7%) to be more frequently affected than females (62/974, 6.4%) (p = 0.05).

**Fig 13:**
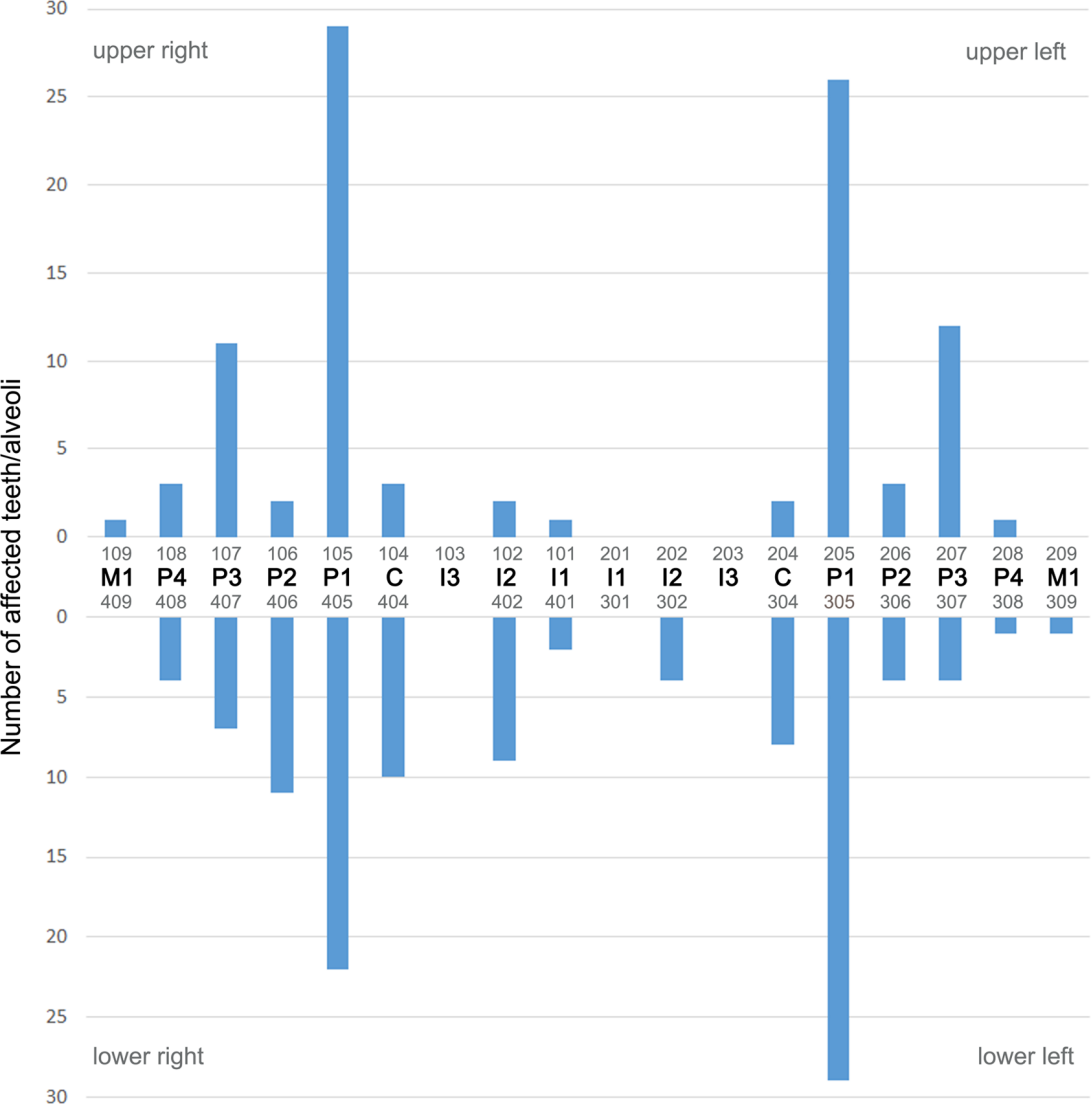
Distribution of periapical lesions visible on external inspection in the permanent dentition of the studied Eastern Atlantic harbor seals (*Phoca vitulina vitulina*). The first premolar was the most frequently affected tooth position. The teeth are identified both by anatomical terms and the numbering of the modified Triadan system (see Fig 1).

Periapical lesions were significantly (p < 0.001) more common in adult (94.4% of affected individuals) than in juvenile/subadult seals (5.6%). No periapical lesions were diagnosed in neonatal/early postnatal animals. The maximum number of periapical lesions diagnosed in an individual was seven. Periapical lesions affected 212 teeth/alveoli, 96 (45.3%) in the maxillary and 116 (54.7%) in the mandibular dental arcade. The first premolar was the tooth position most often affected (n = 106, 50% of recorded cases), followed by the third premolar (n = 34, 16%) and the canine (n = 23, 10.8%) (Fig 13). In four cases, periapical lesions affected two adjacent teeth/alveoli.

### Enamel hypoplasia

No cases of enamel hypoplasia were recorded in the studied teeth.

## Discussion

The present study reports and analyses the occurrence of dental abnormalities and lesions in a large sample of Eastern Atlantic harbor seals, based on the systematic examination of skulls from a scientific collection.

When studying pinniped skulls from museum or other scientific collections for dental or skeletal abnormalities and lesions, it must be considered that a large portion of the material belongs to stranded animals. In a death sample of such origin, pathological conditions are probably overrepresented compared with the general population [18, 55]. However, in the present study about three quarters of the studied skulls originated from seals that were collected during the PDV-epizootic in 1988 that caused the death of about 57% of the population [33]. In harbor seals inoculated with PDV, spontaneous death was observed 11 to 16 days after infection [56]. The high mortality among PDV-infected animals and the rapid death after infection suggests that the prevalence of pathological conditions in our study sample more closely matches the situation in the source population than is the case in most other death samples, as has already been argued in the case of osteoarthritic lesions of the temporomandibular joint [57].

None of the inspected skulls exhibited persistent deciduous teeth, matching previous findings in the Eastern Pacific harbor seal [18]. In our study sample, only a single two-week-old individual with some shed deciduous teeth still attached to soft tissue structures was observed.

Congenital absence (agenesis) of teeth was a relatively rare condition in the Atlantic harbor seal, affecting only 1.4% of the studied animals. Previously, Könemann and van Bree [11] reported a higher frequency of the condition (in 9 of 305 examined individuals, 3.0%, 10 congenitally missing teeth) in a sample of harbor seal skulls from the North Atlantic, held in three collections in the Netherlands. No case of tooth agenesis was found in a sample of 69 harbor seal skulls from museums in the UK, France and the Netherlands [20]. As only few of the skulls from our study sample were radiographed, it cannot be excluded that non-eruption (retention) of a tooth was wrongly classified as a case of agenesis. The actual frequency of tooth agenesis could therefore be somewhat lower than reported. However, also in the other studies reporting frequencies of tooth agenesis in pinnipeds, radiographic analysis was not routinely or not at all performed [11, 13, 16–20, 23]. Therefore, it seems justified to compare the frequencies of this condition among the different study samples.

In our study sample, the number of congenitally missing teeth corresponded to 0.1% of the PTM. The same frequency can be calculated from the data given by Könemann and van Bree [11] for their sample. Aalderink et al. [18] reported a frequency of 0.2% for congenitally missing teeth in the Eastern Pacific harbor seal (*Phoca vitulina richardii*), while for the Californian sea lion (*Zalophus californianus*) Sinai et al. [17] give a value of 0.1%. Higher frequencies (0.6% and 2.8%, respectively) were reported for the Northern fur seal (*Callorhinus ursinus*) [19] and the Northern elephant seal (*Mirounga angustirostris*) [13]. A much higher frequency (44.5% of PTM) of congenitally missing teeth was recently described for the walrus (*Odobenus rosmarus*), with none of the examined skulls having a full complement of maxillary or mandibular teeth [23]. Both in the South American fur seal (*Arctocephalus australis*) [16] and in the Northern fur seal [19], the tooth position most frequently affected by agenesis was the M^2^. This is consistent with the evolutionary trend towards a reduction in the number of molars in otariids [16]. Second molars are not present in the harbor seal dentition. However, a single maxillary second molar has been observed in a harbor seal from the Kattegat [Kahle et al., unpublished observation].

Our finding that tooth agenesis preferentially affected the mandibular dentition parallels the findings by Könemann and van Bree [11] who observed this condition solely in the mandible. In our sample, the tooth position most commonly affected by agenesis was the first premolar, which matches previous findings in this species [11, 18]. The P1 is the smallest premolar in the harbor seal and, as in other extant pinniped species, the tooth at this position is not replaced [8, 38, 45]. According to the histological study of Kubota and Togawa [9] in the Northern fur seal, the first premolar belongs to the permanent dentition, and the primordium of the deciduous P1 undergoes regression early during development. Congenital absence of the P1 will occur if the primordium of the dP1 and the associated portion of the successional dental lamina regress before the latter has given rise to the primordium of the permanent tooth, or if the P1 primordium does not reach the critical size required for continued development [58].

The agenesis of all teeth in a male seal (specimen ZIK 4626) is highly remarkable. The initial brief report [54] also mentions the almost complete lack of hairs in the individual, but did not attempt a diagnosis. We diagnose the condition in the harbor seal as most likely representing a case of ectodermal dysplasia (ED). ED is a term describing a group of hereditary disorders that affect the development of two or more ectodermal structures and cause abnormalities of skin, hair, nails, sweat glands and teeth [59–61].

In humans, the most common subtype of ED is hypohidrotic ED (HED) that is characterized by missing teeth (oligodontia or anodontia), abnormal tooth shape (in the case of oligodontia), partial or complete lack of hair and nails and impaired sweat glands [60, 62]. Most cases of HED are X-linked and caused by mutations in the X-chromosomal *EDA* gene that encodes a ligand (ectodysplasin A1, EDA-A1) belonging to the tumor necrosis factor-α family. HED can, however, also be caused by mutations in autosomal genes (*EDAR*, and *EDARADD*) encoding the EDA-A1 receptor and the adaptor EDARADD involved in the ectodysplasin pathway [63]. HED can furthermore be caused by mutations in the *WNT10A* gene [62, 64]. In the X-linked *Tabby* mutant in mice (*Mus musculus*), mutations in the mouse ortholog of the *EDA* gene cause a phenotype similar to that observed in humans, and X-linked HED has also been confirmed in dogs (*Canis lupus familiaris*) and cattle (*Bos taurus*) [65–68].

To the best of our knowledge, ED has not previously been diagnosed in pinnipeds. The findings in the male harbor seal (specimen ZIK 4626) are basically consistent with a pinniped equivalent of HED, involving complete agenesis of teeth.

In our study sample, 1.1% of the teeth (PTM) were missing due to intravital loss. Previously, a higher value of 2.3% was reported for the Eastern Pacific harbor seal [18]. Lower frequencies of, respectively, 0.4%, 0.7%, and 0.8% were found in the California sea lion [17], the Northern elephant seal [13] and the Northern fur seal [19], while a higher frequency (3.3%) of teeth missing due to intravital loss was recorded in the walrus [23].

In our sample, except for three juvenile/subadult individuals, intravital tooth loss was observed only in adult animals (>5 years). Similarly, for the Eastern pacific harbor seal, Aalderink et al. [18] state that adults exhibited significantly more intravital tooth loss than juveniles and neonates. Given the age dependence of intravital tooth loss, a higher proportion of older individuals in a study sample will likely increase the frequency of the condition.

One or more supernumerary teeth were found in 3.4% of the Eastern Atlantic harbor seals examined by us, compared to a value of 4.6% previously reported for the Eastern Pacific harbor seal [18]. Drehmer et al. [20] recorded no cases of supernumerary teeth in the *Phoca vitulina* skulls (n = 69) studied by them, while Cruwys and Friday [14] found three cases among 140 harbor seals (2.1%). In our study sample, females showed hyperodontia significantly more frequently than males. In contrast, Könemann and van Bree [11], who also studied skulls of Eastern Atlantic harbor seals, found no case among 144 females, compared to 2.1% of the studied males (n = 94).

In our sample, the percentage of individuals with supernumerary teeth (3.4%) was higher than that of animals with congenitally missing teeth (1.4%). This matches previous findings in different phocid species [20, 69]. It has been emphasized that this is opposite to the typical situation in most mammal species, where tooth agenesis is more frequent than presence of supernumerary teeth [20, 69].

While in our sample, most supernumerary teeth were associated with the third and fourth premolars, in the Eastern pacific harbor seal, the first premolar was the tooth position most commonly associated with a supernumerary tooth, while none was observed in association with the third or fourth premolars [18].

Supernumerary teeth are mostly classified as either cases of atavistic (typical) hyperodontia, i.e., the re-occurrence of an element of the dentition that was lost in the course of evolution, or of atypical hyperodontia, if the additional element cannot be related to a tooth present in the evolutionary ancestors of the species under study [7, 20, 70, 71]. The supernumerary teeth observed in our study sample are all considered to represent cases of atypical hyperodontia, resulting from disturbances during dental development, either in the form of the production of additional tooth germs at the dental lamina or the complete splitting of a tooth germ [7, 20, 70].

In the present study, abnormal shapes were observed both in tooth crowns and roots. The case of the abnormally small premolars and molars with simplified (tricuspid) crown shape (specimen ZIK 27251) may be explained on the basis of an etiological model that links variations in tooth size, shape and number [58]. This model predicts that a reduction in tooth size beyond a certain threshold is associated with a simplification of tooth form. The fact that all premolars and molars of the individual were small and exhibited a simplified crown shape strongly suggests a genetic cause of the condition. Based on findings in humans [58], a mutation in the *Pax-9* gene could be a possible candidate in this context.

In our study sample, males were significantly more frequently affected by tooth fractures than females. This is in contrast to the findings by Aalderink et al. [18] in the Eastern Pacific harbor seal, who observed no difference in tooth fracture frequencies between the sexes. While we recorded a significantly higher frequency of tooth fractures in adult compared to juvenile/subadult individuals, this was not the case for the comparison between juvenile and adult Eastern Pacific harbor seals [18]. Generally, a higher frequency of tooth fractures in older compared to younger seals has to be expected, as the probability of fracturing increases with the duration of tooth function. The higher frequency of tooth fractures in male compared to female harbor seals observed in our study sample can be related to the fact that these teeth are not only important for prey capture, but are also used during inter-male competition for females that frequently involves vigorous fighting [31].

With, respectively, 2.1% and 3.6% of the studied individuals affected, the frequency of tooth fractures was low both in the Eastern Atlantic (our study sample) and the Eastern Pacific harbor seal [18]. Much higher frequencies were reported for the walrus (10.5%) [23] and the Northern fur seal (16.6%) [19].

Periapical lesions typically occur as a result of a pulp exposure and subsequent pulp infection, inflammation (pulpitis) and eventual necrosis [72]. From the pulp, the infection spreads into the periapical space, causing a periapical immune response that involves inflammation and related osteoclastic destruction of periapical bone and sometimes also external root resorption [73].

On the basis of macroscopic inspection, periapical lesions can only be diagnosed if they cause visible bone changes, such as an increased number of vascular foramina, focal bone apposition, changes in bone contour or, openings of draining tracts. Initial or subtle periapical lesions, which are not associated with any of the above signs, can only be detected by radiographic examination. Therefore, the periapical lesions recorded in our study sample represent the minimum frequency of this condition.

In our sample, 7.5% of the examined skulls were diagnosed with at least one periapical lesion. For the Eastern Pacific harbor seal, Aalderink et al. [18] reported a much lower frequency of 2.1% affected individuals. Lower frequencies of periapical lesions compared to the Eastern Atlantic harbor seal were also observed in the Northern fur seal (0.7%) [19] and the walrus (3.9%) [23]. Both in the Eastern Atlantic harbor seal (this study) and the Eastern Pacific harbor seal [18] periapical lesions occurred more frequently in adult individuals than in non-adults. The differences in the prevalence of the condition among the two studies could therefore in part be caused by different age composition of the study samples.

The absence of enamel hypoplasia in our study sample matches previous findings in the Eastern Pacific harbor seal [18]. The large sample size in our investigation makes it unlikely that the condition, if it existed, had remained undiagnosed.

In number of seals from our study sample, tooth development had occurred during the PDV-epizootic in 1988. The PDV is closely related to the canine distemper virus (CDV) that belongs to the same genus (*Morbillivirus*) and causes distemper in terrestrial and marine species of Carnivora and also some non-carnivoran species [74, 75]. Among other signs, CDV-infection in dog puppies is known to cause lesions in ameloblasts and other cells of the enamel organ [76, 77]. In individuals that recover from distemper, the permanent teeth whose crowns formed during CDV-infection therefore exhibit enamel hypoplasia [77–79]. Considering the clear link between CDV-infection and enamel hypoplasia in the canine dentition, the lack of enamel hypoplasia in the harbor seals that had grown their teeth in the year of the PDV-outbreak is remarkable.

Detailed data on the timing of the secretory stage and the subsequent maturation stage of amelogenesis in the teeth of the harbor seal are not available. However, given the early formation of the permanent dentition in the species, the secretory stage of amelogenesis must be completed prior to birth in all teeth. If the PDV is capable of exerting similar effects on the enamel organ of seals (not yet demonstrated) as does the CDV in dogs, infection of the seals would therefore have to occur already during the intrauterine stage. However, transplacental transmission of morbilliviruses has thus far not been documented in pinnipeds [80]. In our view, the early development of the dentition in the harbor seal is therefore a key factor that could explain the absence of PDV-related enamel hypoplasia in this species. Moreover, female seals infected with the PDV during pregnancy are prone to abortion [80].

There exist various genetic and environmental (other than the PDV) factors (e.g., hypoplastic type of amelogenesis imperfecta, nutritional disorders, metabolic disturbances, bacterial and viral infections) that can cause enamel hypoplasia in prenatally forming teeth [47, 81–83]. The lack of hypoplastic defects in the enamel of the studied harbor seals is thus a highly interesting observation. Further studies are needed to clarify the reasons for the apparent absence of enamel hypoplasia in the harbor seal.

## Conclusion

The present study found a variety of congenital and acquired dental anomalies and lesions in the studied Eastern Atlantic harbor seals. Differences in the prevalence of certain conditions were observed both between sexes and between age classes (juvenile/subadult vs. adult) as well as between the Eastern Atlantic harbor seal and the Eastern Pacific harbor seal [18]. The occurrence of a presumed case of HED is reported for the first time in a pinniped species. Our findings emphasize the importance of analyzing larger samples of archived skull material from museum collections for obtaining information on the spectrum of dental anomalies and lesions as well as the frequencies of these conditions in wild mammals. This approach has previously been used by us to gain insight into the occurrence and lesional spectrum of temporomandibular joint osteoarthritis in the Eastern Atlantic harbor seal [57]. The study of museum collections can contribute to a better understanding of the health status of marine and terrestrial mammal species, and, in addition, allow the reconstruction of temporal changes in the health condition of populations [84] and comparisons of the health status both among populations within species and between different species. The information from such studies is therefore not only important regarding comparative pathology, but can also be helpful for the management and protection of wild mammals.

## Supporting information

S1 Fig Map showing the provenance of the Eastern Atlantic harbor seals (*Phoca vitulina vitulina*), whose skulls were analyzed in the present study. (a) Wadden Sea of Schleswig-Holstein, (b) Wadden Sea of Hamburg & Elbe Estuary, (c) WaddenSea of Lower Saxony, (d) Heligoland. Basemap adapted from Esri, HERE, DeLorme, MapmyIndia, OpenStreetMap, and the GIS user community. The overview map of Germany is attributed to David Liuzzo under Creative-Commons-Licence.
(TIF)

S1 Table Overview of the Eastern Atlantic harbor seals (*Phoca vitulina vitulina*), whose skulls were analyzed in the present study. (XLSX)
