# Supplementary figures and images for: Dental anomalies and lesions in Eastern Atlantic harbor seals, Phoca vitulina vitulina (Carnivora, Phocidae), from the German North Sea
Source: PLoS One. 2018 Oct 3;13(10):e0204079. doi: 10.1371/journal.pone.0204079 (PMC6169878; doi:10.1371/journal.pone.0204079)

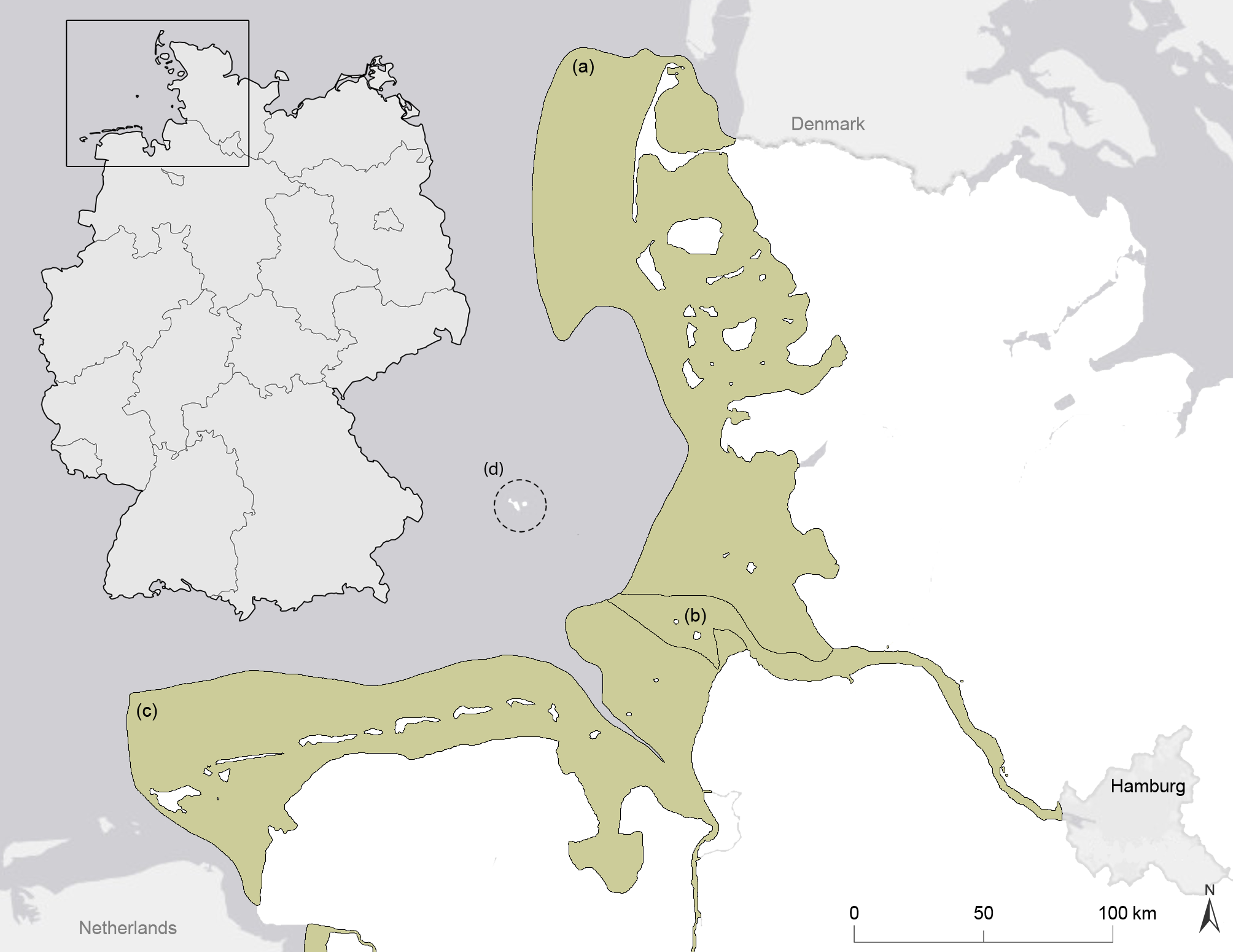

Supplement: S1 Fig — (a) Wadden Sea of Schleswig-Holstein, (b) Wadden Sea of Hamburg & Elbe Estuary, (c) WaddenSea of Lower Saxony, (d) Heligoland. Basemap adapted from Esri, HERE, DeLorme, MapmyIndia, OpenStreetMap, and the GIS user community. The overview map of Germany is attributed to David Liuzzo under Creative-Commons-Licence. (TIF) [file pone.0204079.s001.tif]
